# Are processed fruits and vegetables able to reduce diet costs and address micronutrient deficiencies? Evidence from rural Tanzania

**DOI:** 10.1017/S1368980022000982

**Published:** 2022-09

**Authors:** Jacob Sarfo, Elke Pawelzik, Gudrun B Keding

**Affiliations:** Division Quality of Plant Products, Department of Crop Sciences, University of Goettingen, Goettingen, Germany

**Keywords:** Africa, Diet costs, Fruits and vegetables, Micronutrients, Processed foods, Children and women

## Abstract

**Objective:**

To assess the impact of integrating processed fruits and vegetables (FV) into diets in terms of diet cost reduction and ensuring nutritional adequacy year-round.

**Design:**

Market surveys were conducted to record foods. Focus group discussions (FGD) and 24-h dietary assessments – from children and women – were carried out to determine culturally accepted dietary habits. Six processed FV were considered for addition to diets. Using the Cost of Diet linear programming tool, standards diets were first modelled, and subsequently, the processed FV were included to analyse their impact.

**Setting:**

Rural Tanzania: Mtwara and Morogoro.

**Participants:**

Market survey: 50 traders; FGD: 40 women; 24-h recalls: 36 infants aged 6–23 months, 52 children aged 6–13 years and 292 women.

**Results:**

The standard diet costs between TZS 232 and 2368 (USD 0·3–3) daily for infants. For children aged 6–13 years, it costs between TZS 1711 and 7199 (USD 2·2–9·1) daily and the cost for the women was between TZS 2793 and 10 449 (USD 3·5–13·2). Addition of the processed FV reduced diet costs by up to 61 %, 48 % and 49 % for children aged 12–23 months, children aged 6–13 years and women, respectively. However, for infants aged 6–11 months, costs rose by up to 127 %. The processed FV addressed all micronutrient gaps in the diets except for infants aged 6–11 months, where some micronutrient intakes were unfulfilled.

**Conclusions:**

Processed FV could provide a feasible option to ensure availability of nutritious but cheap diets year-round. Hence, interventions to process FV into nutritious and affordable products should extensively be pursued.

Globally, the number of undernourished people is estimated at 820 million, a figure which has been rising since 2015, particularly in Africa, Latin America and West Asia^(1)^. Currently, 149·2 million children under 5 years of age are stunted and 45·4 million are wasted, while 571 million reproductive-aged women suffer from anaemia^(2)^. In Tanzania, three in five children under 5 years of age and 45 % of women of reproductive age suffer from anaemia^(3)^. About 14 % of school-aged children are anaemic^(4)^. Additionally, 34 %, 5 % and 14 % of the young children are stunted, wasted and underweight, respectively^(3)^. One of the leading drivers for such high and continuously rising malnutrition numbers resulting in mortalities and morbidities is the consumption of poor and unbalanced diets. Most people, particularly the poor, cannot afford healthy diets due to issues of availability and accessibility: availability in the sense that most nutritious foods like fruits and vegetables (FV) are seasonal, in addition to their high perishability rate leading to inadequate supply for consumption year-round^(1,2,5)^. Also, FV are high in price, making them expensive for poor people to buy, which brings in the accessibility factor^(6)^. This phenomenon greatly contributes to food insecurity, where food-insecure households could be trapped in a cycle of poverty^(7)^. Current consumption estimates of FV in Tanzania are below recommended levels: fruit consumption for children between 6 and 23 months and 6 and 13 years is 24 g/d and 74 g/d, while vegetable consumption is 33 g/d and 88 g/d, respectively^(8)^. For women of reproductive age, fruit consumption is 56 g/d and vegetable intake is 120 g/d according to Sarfo *et al.* (unpublished results).

One feasible solution for making FV available and accessible year-round for consumption is the processing of FV. FV can be transformed into products with a longer shelf life, durable, nutritious, convenient and easily transportable for consumers to have easy accessibility^(9,10)^. Through processing – of FV – households can be buffered during food insecurity periods^(7)^ and could contribute substantially to providing quality diets. For instance, consumers cited inconvenience and health-related risks in Tanzania for not consuming regularly fresh cassava leaves despite their nutritional benefits^(11)^, thereby the need for processing. Also, processed FV contribute to increased FV consumption; in the USA, consumers of frozen FV increased their consumption for FV significantly when compared with non-consumers of frozen FV. Nutrient intakes were also higher^(12)^. Notwithstanding these positives, there are negative perceptions associated with processed foods as unhealthy. This, among others, is because the benefits of processed foods, especially the nutritious ones, have not been communicated clearly and consistently to consumers to overcome the negative messaging^(7,13)^. Indeed, not all processed foods contribute to adverse health outcomes, including overweight and obesity^(14)^, and as such, a distinction on them should be unambiguous. Dwyer et al.^(15)^ espoused that processed foods can provide critical micronutrients otherwise unavailable in diets. Moreover, some processed foods can provide valuable nutrients beyond those provided by fresh ones depending on the processing method involved, which could retain and even enhance many nutrients^(10)^.

Yet data or studies on processed foods – particularly FV – in their contribution to quality diets have been limited. Within sub-Saharan Africa and rural areas, studies on them are almost non-existent. The limited data on the contribution of processed foods to diets could contribute to the increasing miscommunication about processed foods and serve as a disadvantage to households, especially for poor households, who may need to rely on them to meet their nutritional needs. Additionally, studies to evaluate their contribution to ensuring inexpensive but nutritious diets are sparse.

Therefore, this study aims to contribute to these gaps in the literature by exploring the impact of processed FV in ensuring nutritional adequacy in diets and reducing nutritious diet costs. In doing so, the present study focused on infants (aged 6–23 months), school-aged children (aged 6–13 years) and women of reproductive age (aged 15–49 years), excluding pregnant women, in rural Tanzania. Specifically, the study sought to answer these two questions: (a) What are the costs and nutrient gaps (if any) in standard diets from local food sources for children and women in rural Tanzania year-round? and (b) Are processed FV able to reduce the standard diet costs and fill nutrient gaps (if any)?

This study is integrated into a larger project named ‘Fruits and Vegetables for all seasons’ (FruVaSe). The project seeks to promote improved resource-efficient processing techniques and new market solutions for surplus FV for rural development in Kenya, Tanzania and Uganda.

## Materials and methods

### Study areas and design

This study was conducted in Tanzania, one of the three study countries under the FruVaSe project. In Tanzania, two regions, namely Mtwara and Morogoro, were selected. Mtwara is in the southern part of Tanzania, with rainfall patterns between 840 and 1250 mm. The region is predominantly rural, with 80 % of the population engaged in farming^(3)^. Mtwara is also one of Tanzania’s largest cashew production areas^(16)^. Morogoro is situated in the eastern part of Tanzania and is the third largest region in terms of land size. Annual rainfall is projected between 600 and 1200 mm. Just as Mtwara, the region is predominantly rural. In both regions, agriculture constitutes the main economic activity for most of the population^(3)^.

First, the two regions – Mtwara and Morogoro – were purposively selected under the FruVaSe project to study cashew apple and African nightshade, respectively. These FV were selected because they are considered nutritious yet underutilised. Again, through purposive sampling, two districts within each study region were chosen because of their cultivation and subsequent availability of the two crops in the selected sites. In Mtwara, Tandahimba and Masasi districts were selected, while Kilombero and Morogoro DC districts were chosen in Morogoro. Communities within these districts were compiled, and five communities were randomly selected per district. A list of households from the selected communities was collected with the help of community health and nutrition workers and community leaders. From the list, thirty households were randomly selected per community in Mtwara. While in Morogoro, the randomly selected households were between 20 and 47 households per community, which was proportional to the number of households. Overall, 300 households were sampled per study area. A detailed description of the design for Tanzania, including the inclusion and exclusion criteria for compiling household lists, has been published elsewhere^(14)^.

### Data collection

Data were collected across the plenty and lean seasons of cashew apple and African nightshade in Mtwara and Morogoro, respectively. In Mtwara, data collection was conducted in October/November 2019 for the plenty season and April/May 2019 for the lean season. Surveys for plenty and lean seasons in Morogoro were performed in June/July 2019 and January 2020, respectively.

Market surveys across the two seasons were carried out in the ten communities selected per study region. Four traders selling assorted food items were sampled in each community, totalling forty traders each for Mtwara and Morogoro. However, not all traders were met during both seasons. Some traders had either closed their shops or had moved or could not be identified despite our confirmation with them for a second survey during the initial survey. In Mtwara, thirty-three traders were surveyed during both seasons, while it was seventeen traders in Morogoro. During the survey, all food prices and their corresponding weights were collected at which they were sold. Three different samples of all foods were weighed that corresponded to the same price to ensure accuracy, except for packaged foods with weights printed on them.

Next, all foods recorded from the market survey were compiled for a focus group discussion (FGD) to determine culturally accepted dietary habits. The FGD was one of two tools used to construct dietary behaviour. In doing so, women were recruited using the following criteria: first, they were part of the sampled households; second, they prepared and served meals and acted as caregivers for children; and third, they were willing to participate and provide sufficient information. In each study region, two FGD were conducted, consisting of ten women each, two from each selected community. The FGD in Mtwara was conducted during the plenty season, while Morogoro was conducted during the lean season; however, the discussion was not related to the current season. During the FGD, the women answered questions about the frequencies of food intake by selecting one of these options for each food compiled: usually (5+ d/week), often (1–4 d/week), rarely (once a month or year) and never. They were further asked to identify foods considered taboo or are not eaten in the communities.

The second dietary behaviour tool employed was the 24-h dietary assessment. Four non-consecutive dietary assessments – two per season – were conducted across the sampled households for the women. Two non-consecutive assessments were carried out for the children – one per season. In each recall survey, the women identified all foods and drinks consumed during the previous 24 h of the survey day for themselves and their infant children. The school-aged children identified for themselves the foods and drinks consumed. Each individual’s frequency of foods and drinks consumption was constructed into scores (see data analysis) to represent dietary behaviour from the 24-h dietary assessment.

### Processed fruits and vegetables

The processed FV used in this study could be classified as minimally processed because the mode of processing includes processes such as drying and freezing that do not drastically alter the nutrients of the FV. In all, three processed fruits and three processed vegetables were used, namely cashew apple juice (CAJ), guava-cashew nut bar (GCB), jackfruit-cashew nut bar (JCB), as well as dried African nightshade (DAN), cassava leaves powder (CLP) and dried cowpea leaves (DCL). All six products were developed within the FruVaSe projects. However, nutrient content data were already available for only two products – the guava and jackfruit cashew nut bars. The nutrient contents of the remaining products were compiled from literature through Google Scholar search. In Table 1, the nutrient profiles of the processed FV have been compiled on a fresh weight basis per 100 g edible portion. Bioavailability rates of 5 % and 48 % were used to convert Fe and Ca into absorbed quantities^(17)^. Additionally, β-carotene amounts were converted into retinol equivalent using the ratio 12:1^(17,18)^. All six FV products are described in the following:

**Table 1:** Nutrient composition and prices (in Tanzania shillings (TZS) and USD ($)) of the processed fruits and vegetables per 100 g of edible portion added to the modelled diets for women and children in Mtwara and Morogoro, Tanzania

Processed fruits and vegetables	Price in TZS	Price in USD ($)	Energy (kcal)	Fat (g)	Protein (g)	Fibre (g)	Vitamin A (μg)*	Vitamin C (mg)	Fe (mg)*	Zn (mg)	Ca (mg)*	Thiamine (μg)	Riboflavin (μg)	Na (mg)	Phosphorous (mg)	Potassium (mg)	Mg (mg)
Cashew apple juice	163	0·2	n.a	n.a	n.a	n.a	n.a	252·4	0·07	0·03	60·9	n.a	n.a	0·3	0·9	86·4	12·8
Guava-cashew nut bar	364	0·5	n.a	n.a	n.a	n.a	n.a	n.a	5·8	6·00	71·9	n.a	n.a	41·3	385·4	1034·1	163·1
Jackfruit-cashew nut bar	364	0·5	n.a	n.a	n.a	n.a	n.a	n.a	4·8	6·2	89·0	n.a	n.a	40·2	347·7	1128·2	164·3
Dried African nightshade	552	0·7	258·4	1·5	26·6	19·2	1926·0	38·7	n.a	n.a	n.a	0·5	1·8	n.a	n.a	n.a	n.a
Dried cassava leaves powder	552	0·7	251·5	5·4	26·5	14·1	229·2	45·2	13·5	9·8	609·7	4·8	n.a	35·4	280·9	1324·6	273·9
Dried cowpea leaves	552	0·7	124·7	n.a	31·2	14·2	1836·8	122·8	0·5	0·1	22·4	n.a	n.a	1426·9	30·0	n.a	n.a

* Ca and Fe were converted into absorbed quantities using the rates 48 % and 5 %, respectively; vitamin A is retinol equivalent converted from β-carotene using the ratio 1:12.

n.a: Not available in literature/not determined.

Sources are from refs 19–25.

*Cashew apple juice*: the cashew apple was first macerated and the juice was extracted from it. The juice was refrigerated and frozen at –20°C for nutrient analyses^(19)^, as presented in Table 1. *Guava-cashew nut bar and jackfruit-cashew nut bar*: the guava bar consisted of guava with some mango, cashew nuts, desiccated coconut and lemon juice, while the jackfruit bar consisted of the same ingredients as the guava bar except that the main ingredient, guava, was replaced with jackfruit. In processing both fruit bars, simple cooking and drying methods were used, which could be suitable and straightforward methods for households and small food processing groups. The fruit nut bars contain high Fe, Zn, Ca (Table 1) and low sugar. Sensory evaluation showed an acceptability trend for the products^(20)^.

*Dried African nightshade*: average nutrient contents of three DAN from three studies were used. All three vegetables were processed through solar drying in temperatures range of 37–63°C^(21–23)^. *Cassava leaves powder*: the cassava leaves were pressed to remove excess water and then heat-treated in a hot dry pot, after which they were pounded and cooked in water. The leaves were then dried at 60°C, milled and stored in airtight ziplock bags^(24)^. *Dried cowpea leaves*: cowpea leaves were harvested and solar-dried until the leaves became brittle when felt in hand. The leaves were then sealed and stored in airtight polythene^(25)^; nutrient contents are depicted in Table 1.

In costing the price of the processed FV, prices of similar products from elsewhere were adopted. Average market prices for snacks and fruit juice in the study regions, which equalled TZS 364 (USD 0·5)/100 g and TZS 163 (USD 0·2)/100 ml, were chosen for the fruit bars and CAJ, respectively. For the processed vegetables, a price of TZS 552 (USD 0·7)/100 g, equivalent to the cost of processed vegetables from a recent study by Tepe et al. (unpublished results), was used.

### Cost of Diet linear programming tool

The Cost of Diet (CoD) linear programming tool was adopted to model standard diets and assess the impact of the processed FV in the diets. The CoD tool is a mathematical linear computer programming software that models diets that meet individuals or a family’s average energy needs as well as their recommended protein, fat and micronutrient intakes at the least financial cost^(17,18,26)^. The CoD tool contains nutrient contents of 3580 food items and supplements extracted from five different food consumption tables. It includes macro- and micronutrient recommendations for different individuals based on the specifications from the WHO and FAO as well as typical portion sizes for different individuals^(17,26)^.

To model diets, additional data upload was needed, including local foods and their corresponding prices and weights and ‘food constraints’ to factor in local food culture. Food constraints – classified into minimum and maximum – refer to the number of times a week that foods can be consumed. Minimum constraints are the least number of times foods are consumed per week and hence can be included in the models, while maximum constraints indicate the maximum number of times foods are consumed weekly^(18,26)^. The present study collected data on local foods and food constraints (see data collection) and used the food tables and portion sizes – minimum and maximum – embedded in the tool for analysis.

### Data analysis

First, the food items and their corresponding prices and weights obtained from the market surveys were entered into the CoD tool. Average prices of the foods per 100 g of edible portion were calculated. The foods were also classified into eight food groups: cereals, roots and tubers; fruits; vegetables; oils and fats; spices and condiments; sweets and beverages; animal source foods; and pulses and nuts. Then, the FGD data on dietary habits were entered to calculate the minimum and maximum constraints of the foods (see online supplementary material, Supplemental Tables S1 and S2). Using average food prices and the FGD food constraints, diets were modelled individually for infants: aged 6–8 months, aged 9–11 months, aged 12–23 months; school-aged children 6–13 years; and reproductive-aged women 15–49 years: non-lactating and non-pregnant and lactating, for both Mtwara and Morogoro.

Using the data from the 24-h recalls, food constraints were constructed to validate the constraints from the FGD. First, the frequency of each food consumption across the dietary recalls was weighted. For example, if a woman consumed tea during all recall periods (four times), a score of 2 was given, thrice was scored 1·5 while twice, once and zero attracted weights of 1, 0·5 and 0, respectively. For both children groups, weights were provided based on the two recalls, and hence, tea consumption for a child during both recalls received a weight of 1, once had a score of 0·5 and no consumption received a score of 0. Each score level per food was multiplied by the respective number of women or children who consumed such food. They were then summed up to obtain the total score per food. Based on the respective overall score, the minimum and maximum constraints (number of times per week a food is consumed) were computed for each food accordingly to set benchmarks described in Supplemental Table S3. After excluding some households due to unavailability during all recall periods, final samples used in Mtwara for the 24-h food constraints were 12 infants, 11 school-aged children and 109 women. For Morogoro, the samples were 24 infants, 41 school-aged children and 183 women. The 24-h food constraints were then entered into the CoD tool to recalibrate the constraints from the FGD and re-model the standard diets. Daily diet costs were calculated for the individuals from the two study sites, in Tanzania Shillings (TZS) and US dollars (USD), using the 2020 purchase power parity for private consumption rate in Tanzania as published by the World Bank^(27)^.

**Fig. 1:**
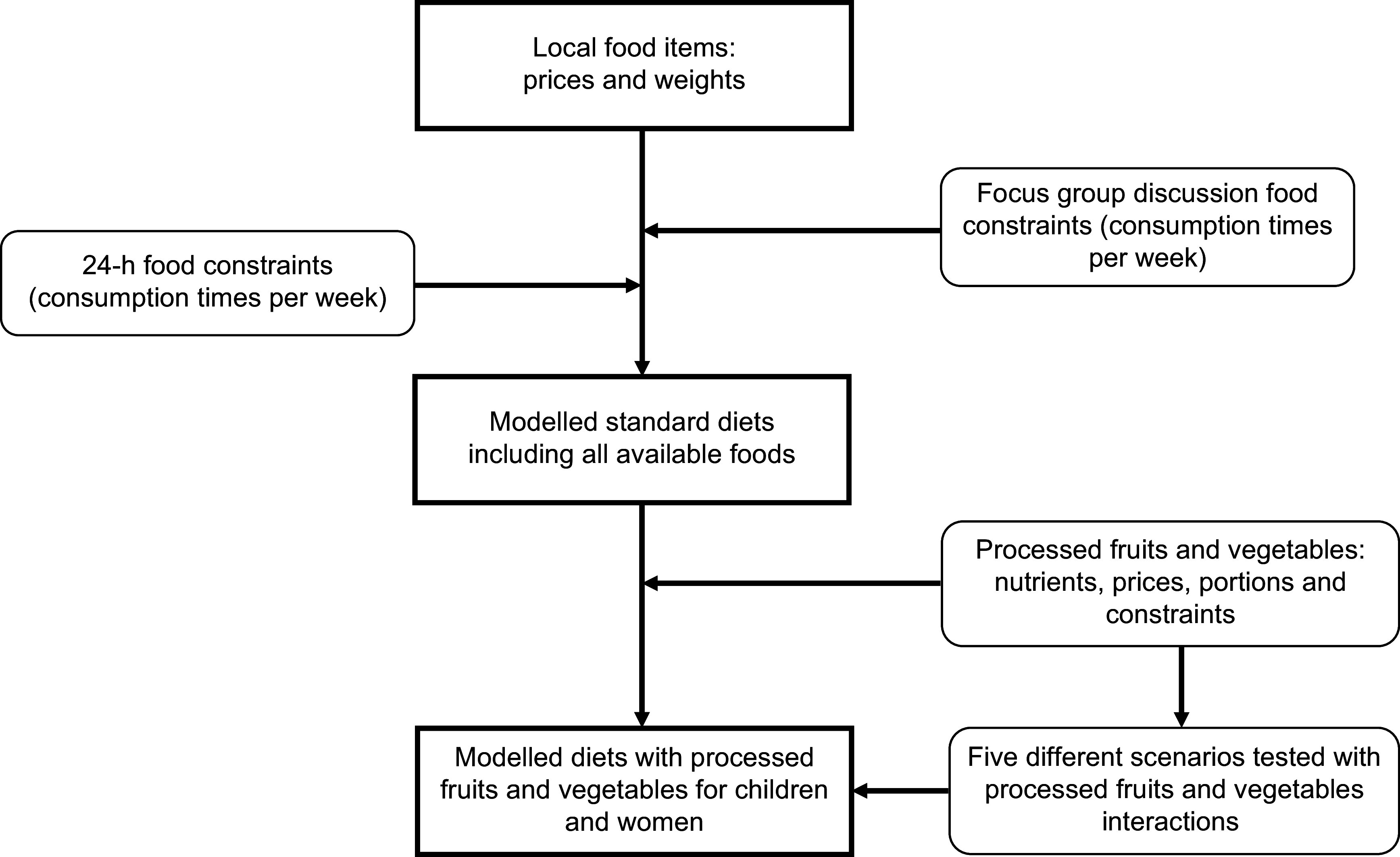
Flow chart of how standard diets were modelled with and without the inclusion of processed fruits and vegetables for children and women in Mtwara and Morogoro, Tanzania

The processed FV, including their nutrients and prices, were added to the models under different scenarios. For the first scenario, minimum and maximum food constraints of zero and seven were set for the processed FV, reflecting the food constraints for most of the FV identified from the 24-h recalls. Additionally, these food constraints provided the CoD tool the free hand to include which processed FV – whether single or by different combinations – impact the diets of children and women in terms of cost and nutrients. Under four other scenarios using food constraints of one and seven (days per week food can be consumed), the CoD tool was forced to select the processed FV: (a) one by one; (b) all processed fruits together; (c) all processed vegetables together and (d) all processed FV together. The first scenario – adding all six processed FV to the models (under zero and seven food constraints) – provided the optimal impact (cost and nutrient) for both children and women in Mtwara and Morogoro. Hence, this scenario was used for further analysis. Finally, the impact of the processed FV in reducing diet cost and bridging nutrient gaps for one day was assessed and represented in percentages. A flow chart of the modelling steps as described earlier has been depicted in Fig. 1.

## Results

### Standard diet cost

Overall, sixty-two food items were identified on the market from thirty-three traders in Mtwara. Most of the foods were from the animal source and sweets and beverages food groups. The animal source food group costs on average – for both seasons – TZS 1366 (USD 1·7)/100 g, while the sweets and beverages food group costs TZS 545 (USD 0·7). Prices for single-food items and average prices for all other food groups are shown in Supplemental Table S4. In Morogoro, seventy-five foods were surveyed from the seventeen traders on the market. Most foods were from the food groups vegetables, fruits, and sweets and beverages as shown in Supplemental Table S5, where other food groups are also listed, including their prices.

The cost of a standard daily diet in Mtwara for children aged between 6 and 23 months was between TZS 301 and 2368 (USD 0·4–3·0) in the plenty season (August–December) and TZS 232 and 1515 (USD 0·3–1·9) in the lean season (January–July). The diet costs TZS 7199 (USD 9·1) and TZS 6207 (USD 7·9) for the school-aged children in the plenty and lean seasons, respectively (Table 2). As shown in Table 2, it would cost between TZS 10 005 and 10 449 (USD 12·7–13·2) and TZS 7448 and 8457 (USD 9·4–10·7) for women to access standard diets in Mtwara during the plenty and lean seasons. The diet cost in Mtwara was mainly driven by the high prices of food sources from the animal source group. On the other hand, diet cost in Morogoro was relatively low. It costs between TZS 346 and 639 (USD 0·4–0·8) in the plenty season (June–November) and between TZS 329 and 670 (USD 0·4–0·9) in the lean season (December–May) to afford the standard diets for infants. The diet cost for school-aged children in Morogoro was about four times less than the diet cost for the same age group in Mtwara in plenty and lean seasons (Table 3). Also, in Morogoro, women would need to pay three times less the price of standard diets for women in Mtwara for both seasons. The modelled diets in Morogoro consisted of more vegetables and animal source foods, and both food groups contributed to a large share of the costs (Table 3).

**Table 2:** Cost of standard diet (in Tanzania shillings (TZS) and US dollars (USD)) for 1 d without processed fruits and vegetables for children and women in Mtwara, Tanzania

Individual	Plenty season			Lean season		
	Food group and contribution to diet cost	Cost in TZS per d	Cost in USD per d	Food group and contribution to diet cost	Cost in TZS per d	Cost in USD per d
6–8 months	Cereals, roots and tubers: 22·0 %	301	0·4	Cereals, roots and tubers: 20·9 %	232	0·3
	Fruits:1·7 %			Fruits: 0 %		
	Vegetables: 25·7 %			Vegetables: 26·3 %		
	Oils and fats: 4·8 %			Oils and fats: 6·4 %		
	Spices and condiments: 0·5 %			Spices and condiments: 0·5 %		
	Sweets and beverages: 2·0 %			Sweets and beverages: 2·5 %		
	Animal source foods: 43·3 %			Animal source foods: 43·3 %		
	Pulses and nuts: 0 %			Pulses and nuts. 0 %		
9–11 months	Cereals, roots and tubers: 11·1 %	658	0·8	Cereals, roots and tubers: 5·0 %	1098	1·4
	Fruits: 12·7 %			Fruits: 0 %		
	Vegetables: 29·8 %			Vegetables: 25·5 %		
	Oils and fats: 2·4 %			Oils and fats: 1·5 %		
	Spices and condiments: 0·2 %			Spices and condiments: 0·1 %		
	Sweets and beverages: 1·0 %			Sweets and beverages: 0·6 %		
	Animal source foods: 42·7 %			Animal source foods: 67·2 %		
	Pulses and nuts: 0 %			Pulses and nuts: 0 %		
12–23 months	Cereals, roots and tubers: 3·9 %	2368	3·0	Cereals, roots and tubers: 4·4 %	1515	1·9
	Fruits: 3·3 %			Fruits: 0 %		
	Vegetables: 10·4 %			Vegetables: 22·5 %		
	Oils and fats: 0·8 %			Oils and fats: 1·3 %		
	Spices and condiments: 0·1 %			Spices and condiments: 0·1 %		
	Sweets and beverages: 0·3 %			Sweets and beverages: 8·7 %		
	Animal source foods: 79·6 %			Animal source foods: 60·6 %		
	Pulses and nuts: 1·5 %			Pulses and nuts: 2·3 %		
6–13 years	Cereals, roots and tubers: 5·0 %	7199	9·1	Cereals, roots and tubers: 5·6 %	6207	7·9
	Fruits:3·4 %			Fruits: 0 %		
	Vegetables: 10·9 %			Vegetables: 15·7 %		
	Oils and fats: 29·1 %			Oils and fats: 39·5 %		
	Spices and condiments: 0 %			Spices and condiments: 0 %		
	Sweets and beverages: 2·9 %			Sweets and beverages: 4·7 %		
	Animal source foods: 47·7 %			Animal source foods: 34·4 %		
	Pulses and nuts: 1·0 %			Pulses and nuts: 0 %		
Women	Cereals, roots and tubers: 2·6 %	10 005	12·7	Cereals, roots and tubers: 2·4 %	7448	9·4
	Fruits:1·3 %			Fruits: 0 %		
	Vegetables: 10·9 %			Vegetables: 19·3 %		
	Oils and fats: 1·3 %			Oils and fats: 1·7 %		
	Spices and condiments: 0·1 %			Spices and condiments: 0·1 %		
	Sweets and beverages: 3·4 %			Sweets and beverages: 7·6 %		
	Animal source foods: 79·4 %			Animal source foods: 67·8 %		
	Pulses and nuts: 1·0 %			Pulses and nuts: 1·2 %		
Lactating women	Cereals, roots and tubers: 2·7 %	10 449	13·2	Cereals, roots and tubers: 3·1 %	8457	10·7
	Fruits: 3·3 %			Fruits:1·0 %		
	Vegetables: 12·2 %			Vegetables: 14·5 %		
	Oils and fats: 1·3 %			Oils and fats: 1·6 %		
	Spices and condiments: 0 %			Spices and condiments: 0 %		
	Sweets and beverages: 6·4 %			Sweets and beverages: 9·0 %		
	Animal source foods: 72·9 %			Animal source foods: 69·4 %		
	Pulses and nuts: 1·3 %			Pulses and nuts: 1·4 %		

$1 = Tsh 790·5 PPP for private consumption.

**Table 3:** Cost of standard daily diet (in Tanzania shillings (TZS) and US dollars (USD)) for 1 d without processed fruits and vegetables for children and women in Morogoro, Tanzania

Individual	Plenty season			Lean season		
	Food group and contribution to diet cost	Cost in TZS per d	Cost in USD per d	Food group and contribution to diet cost	Cost in TZS per d	Cost in USD per d
6–8 months	Cereals, roots and tubers: 14·3 %	346	0·4	Cereals, roots and tubers: 18·5 %	329	0·4
	Fruits: 0 %			Fruits: 0 %		
	Vegetables: 68·1 %			Vegetables: 61·7 %		
	Oils and fats: 4·2 %			Oils and fats: 3·5 %		
	Spices and condiments: 0·3 %			Spices and condiments: 0·4 %		
	Sweets and beverages: 3·5 %			Sweets and beverages: 4·1 %		
	Animal source foods: 9·6 %			Animal source foods: 11·8 %		
	Pulses and nuts: 0 %			Pulses and nuts: 0 %		
9–11 months	Cereals, roots and tubers: 8·3 %	654	0·8	Cereals, roots and tubers: 11·6 %	582	0·7
	Fruits: 0 %			Fruits: 0 %		
	Vegetables: 52·6 %			Vegetables: 41·6 %		
	Oils and fats: 2·5 %			Oils and fats: 2·2 %		
	Spices and condiments: 0·3 %			Spices and condiments: 0·4 %		
	Sweets and beverages: 2·0 %			Sweets and beverages: 2·6 %		
	Animal source foods: 34·3 %			Animal source foods: 41·7 %		
	Pulses and nuts: 0 %			Pulses and nuts: 0 %		
12–23 months	Cereals, roots and tubers: 10·7 %	639	0·8	Cereals, roots and tubers: 12·6 %	670	0·9
	Fruits: 0 %			Fruits: 0 %		
	Vegetables: 35·7 %			Vegetables: 29·1 %		
	Oils and fats: 3·1 %			Oils and fats: 2·4 %		
	Spices and condiments: 0·3 %			Spices and condiments: 0·3 %		
	Sweets and beverages: 2·6 %			Sweets and beverages: 2·8 %		
	Animal source foods: 35·4 %			Animal source foods: 38·5 %		
	Pulses and nuts: 12·2 %			Pulses and nuts: 14·3 %		
6–13 years	Cereals, roots and tubers: 26·7 %	1711	2·2	Cereals, roots and tubers: 32·5 %	1759	2·2
	Fruits:10·7 %			Fruits:1·6 %		
	Vegetables: 44·6 %			Vegetables: 50·4 %		
	Oils and fats: 5·9 %			Oils and fats: 8·5 %		
	Spices and condiments: 0·1 %			Spices and condiments: 0·1 %		
	Sweets and beverages: 0 %			Sweets and beverages: 2·8 %		
	Animal source foods: 11·0 %			Animal source foods: 7·0 %		
	Pulses and nuts: 1·0 %			Pulses and nuts: 0 %		
Women	Cereals, roots and tubers: 18·7 %	2822	3·6	Cereals, roots and tubers: 19·4 %	3100	3·9
	Fruits: 0 %			Fruits: 0 %		
	Vegetables: 37·4 %			Vegetables: 41·7 %		
	Oils and fats: 6·2 %			Oils and fats: 4·6 %		
	Spices and condiments: 0·1 %			Spices and condiments: 0·1 %		
	Sweets and beverages: 1·6 %			Sweets and beverages: 2·1 %		
	Animal source foods: 30·5 %			Animal source foods: 27·2 %		
	Pulses and nuts: 5·6 %			Pulses and nuts: 4·9 %		
Lactating women	Cereals, roots and tubers: 24·8 %	2793	3·5	Cereals, roots and tubers: 25·6 %	3038	3·8
	Fruits: 0 %			Fruits: 0 %		
	Vegetables: 37·7 %			Vegetables: 47·6 %		
	Oils and fats: 8·5 %			Oils and fats: 6·2 %		
	Spices and condiments: 0·1 %			Spices and condiments: 0·1 %		
	Sweets and beverages: 1·6 %			Sweets and beverages: 2·1 %		
	Animal source foods: 19·8 %			Animal source foods: 10·9 %		
	Pulses and nuts: 7·5 %			Pulses and nuts: 7·5 %		

$1 = Tsh 790·5 PPP for private consumption.

### Impact of the processed fruits and vegetables on standard diet costs

**Fig. 2:**
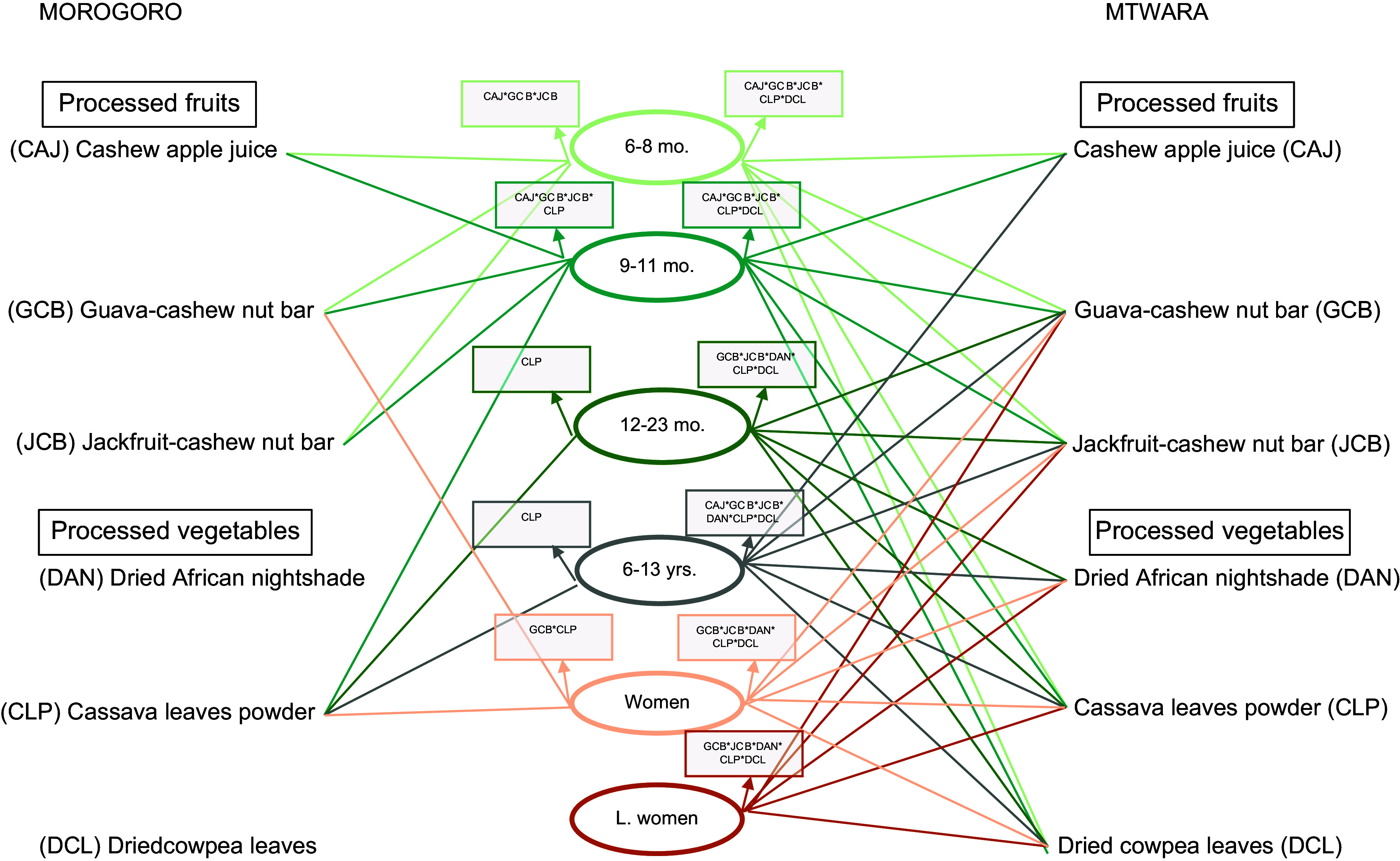
The different interactions of the processed fruits and vegetables that impact costs and nutrients of the standard diets for children and women in Mtwara and Morogoro, Tanzania, year-round

**Fig. 3:**
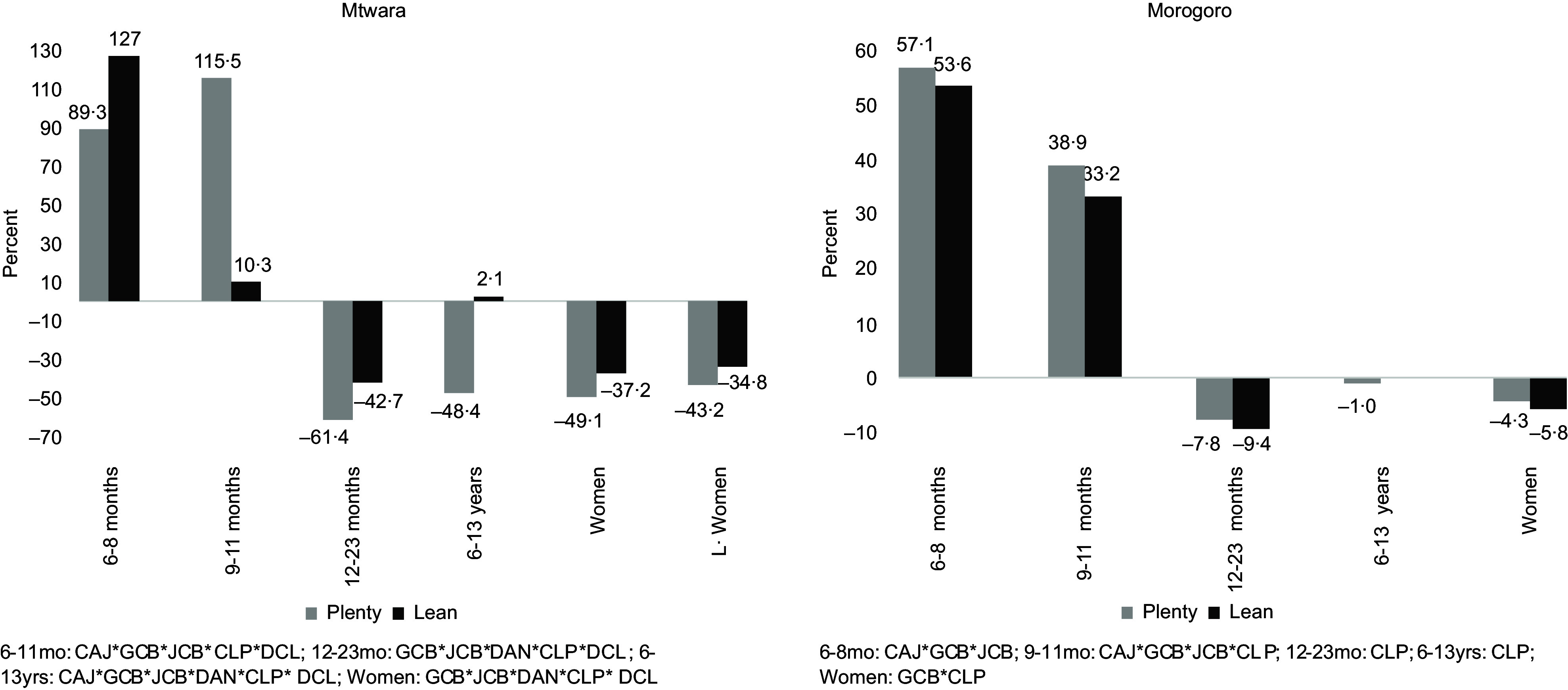
Percentage of reduction/increase in the standard diet cost after integrating processed fruits and vegetables into the models for children and women in Mtwara and Morogoro, Tanzania

The addition of the processed FV to the models showed varying interactions that impacted cost and nutrients across the two study sites and for the individuals. In Mtwara, all three processed fruits – CAJ, GCB and JCB – and two processed vegetables – CLP and DCL – were included in the diets of infants aged 6–11 months (Fig. 2). This increased the diet cost on average by 102 % and 69 % in the lean and plenty seasons (Fig. 3). With a different combination for infants aged 12–23 months: GCB, CLP and DAN in the plenty season, and the two fruits nut bars and all three processed vegetables in the lean, standard diet costs were reduced by 61 % and 43 %, respectively (Fig. 3). Furthermore, all six processed FV added up to the diet of school-aged children in Mtwara except in the lean season where only two were added – GCB and DCL. This culminated in the diet cost reduction by 48 % in the plenty season and slightly going up by 2 % in the lean season (Fig. 3). For the two women groups, diet costs were reduced by an average of 46 % and 36 % in plenty and lean seasons, respectively (Fig. 3). This stemmed from combining the three processed vegetables and the two fruit nut bars to their diets, as indicated in Fig. 2.

The processed FV combinations and their magnitude of impact differed in Morogoro, although the trend is the same as Mtwara. For infants aged 6–11 months, the three processed fruits plus CLP were added to the standard diet, which increased the diet cost, on average, by 48 % and 43 % in plenty and lean seasons, respectively. Only CLP added up to the diet of infants aged 12–23 months (both seasons), the school-aged children and non-pregnant, non-lactating women (only in the plenty season), as shown in Fig. 2. The corresponding proportions of reduction in the cost of standard diets are depicted in Fig. 3. None of the processed FV were included in the diet of lactating women, which meant that the standard diet without the processed FV was adequately cost-optimised and nutritious.

### Micronutrient gaps and the impact of the processed fruits and vegetables

**Fig. 4:**
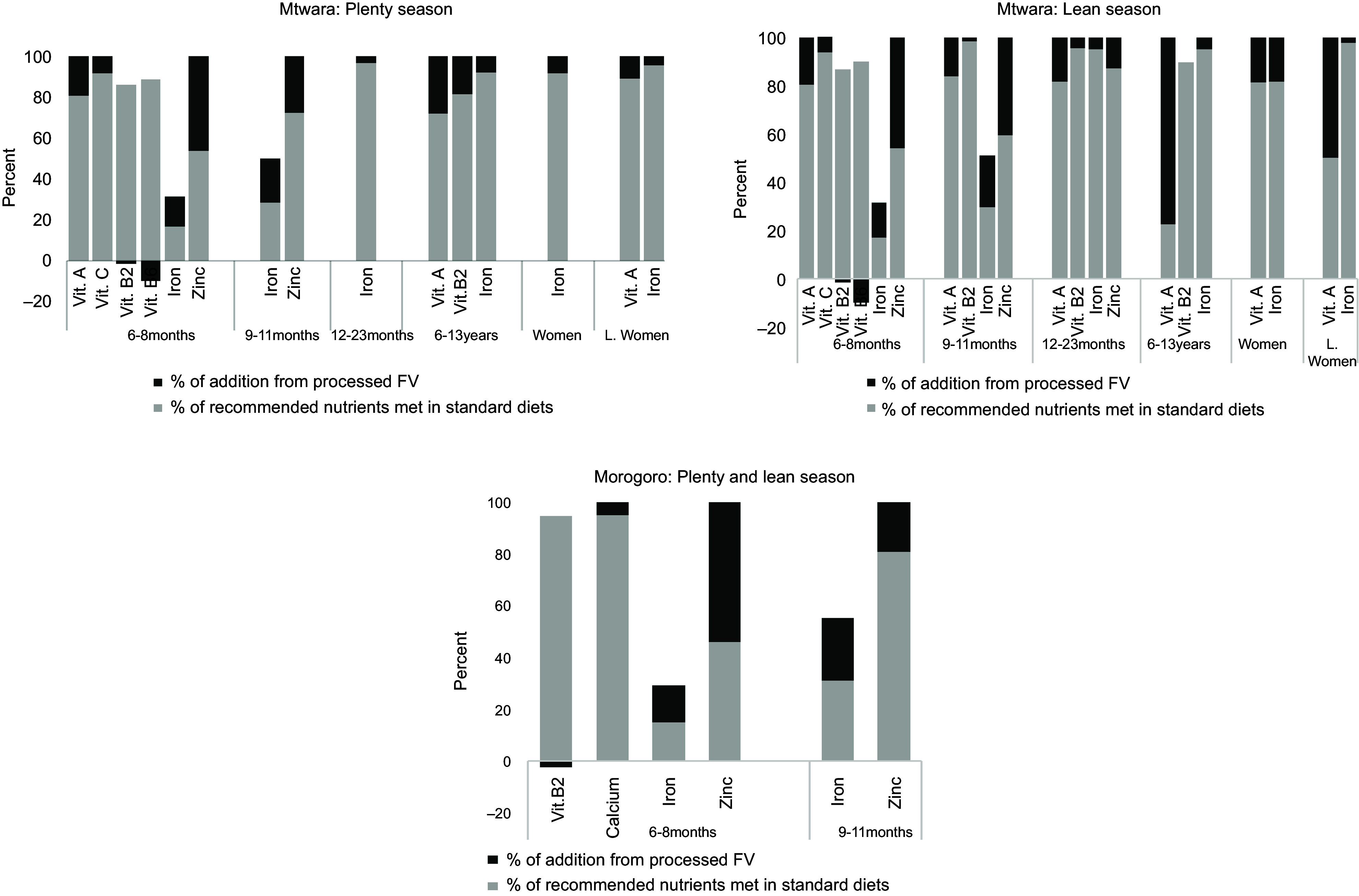
Percentage of recommended nutrients met in the standard diets and after adding the processed fruits and vegetables in the models for children and women in Mtwara and Morogoro, Tanzania

The standard diets showed gaps in micronutrient intakes, particularly for the individuals in Mtwara. While micronutrient deficiencies were found in the diet of only infants aged 6–11 months in Morogoro, all individuals in Mtwara did not meet recommended levels of certain micronutrients in their diets. Vitamin A and Fe were the lacking micronutrients in diets that cut across for all individuals in Mtwara. Additional limited micronutrients included vitamins C, B_2_, B_6_ and Zn for infants aged 6–11 months. For infants aged 12–23 months and school-aged children, vitamin B_2_ was inadequate, in addition to the lack of vitamin A and Fe (Fig. 4). The inclusion of the processed FV at different interactions and combinations – as described above – improved some micronutrient contents. Recommended intakes for vitamins A and C and Zn were met for infants aged 6–11 months; there was a slight increase in Fe content by 18 % and slight reductions in vitamins B_2_ and B_6_ (Fig. 4). The processed FV filled up all recommended micronutrient intakes not met in the standard diets for the rest of the individuals except for the school-aged children, where vitamin B_2_ decreased by 0·5 % in the lean season. In Morogoro, the most pronounced limited micronutrients were vitamin B_2_, Ca, Fe and Zn identified in the diets of infants aged 6–11 months for both seasons. The addition of the processed FV, given by the different combinations as shown in Fig. 2, fully bridged the micronutrient gaps of Ca and Zn. Fe content increased by 19 % on average, while there was a slight reduction in vitamin B_2_, as mapped in Fig. 4.

## Discussion

### Diets and micronutrient deficiencies

In the present study, standard diets were modelled with processed FV to investigate their impact on diet cost and micronutrient intakes. The costs of the modelled diets were primarily driven by vegetables and animal source foods, which indicates the high prices of these nutritious foods. In an EAT-Lancet report^(6)^, where they also modelled diets, they showed that the high cost of FV and animal source foods is a major contributing factor to expensive nutritious diets and the inability for the poor to afford. Also, in Ethiopia, high price levels for FV and animal food sources were likely to prevent the poor from accessing these foods and, consequently, nutritious diets^(28)^. This is a highly likely situation that could exist in rural Tanzania where most people are poor.

Aside from the cost, micronutrient gaps were found in the modelled standard diets. Inadequate micronutrient intakes from diets have been highlighted strongly in earlier studies pertaining to sub-Saharan Africa. In northern Kenya, Fe and Zn were the most limiting nutrients in children’s and women’s diets^(17)^. Again, in Kenya, diets from Baringo lacked Fe, Zn, Ca and vitamin B_6_^(18)^. Additionally, in Mozambique, Fe and vitamin B_2_ were limiting nutrients in diets^(29)^, while Fe, Ca, vitamins B_2_, B_6_ and B_12_ were the micronutrient gaps in diets in Burkina Faso^(30)^. All the micronutrient gaps listed above were also found in the modelled diets in rural Tanzania. Even after integrating the processed FV, vitamins B_2_ and B_6_ and Fe were still limiting for infants aged 6–11 months, underscoring the urgent need to address these micronutrient inadequacies for better nutrition and human development. These nutrient gaps persist partly because of the sources of foods that characterise diets, especially in rural areas. Diets largely consist of plant-based foods with low absorption rates for nutrients such as Fe and Zn^(17,31,32)^. Another contributing factor is the insufficient supply and consumption of micronutrient-dense foods such as FV, meat and dairy foods, largely due to their seasonality and/or high perishability levels. Hence, their inclusion in diets becomes a challenge even if households can afford them. For instance, in this study, the availability of fruits on the market was low, most likely leading to low consumption and limited inclusion in the modelled diets. In addition, low intake of animal source foods, including meat and dairy foods, accounts for the reduction in vitamin B_2_ for infants across locations and seasons, even after adding the processed FV, as these animal source foods are rich in vitamin B_2_^(33)^. Only one animal source food – fish – was included in the diet, emphasising the need to promote sustainable consumption of animal source foods and/or adding needed micronutrients to foods through fortification.

### Nutritional contribution and cost of processed fruits and vegetables

Although most fruits are consumed fresh, compared with vegetables that need to be processed most times before consumption, processing adds the layer of safety and prolonged shelf life. Through processing, anti-nutrients and toxic substances can be inactivated or destroyed to ensure palatability and digestibility. Even though nutrient losses may occur through processing, varied processing techniques can also be used to fortify and/or alter FV with nutrients that are consistent with nutrition guidelines.^(10,13,34)^. This study has shown that processed FV can promote nutritional adequacy in diets and even reduce diet costs for specific individuals. These findings are also backed by Weaver et al.^(13)^, who showed in their study that processed foods could contribute to ensuring food and nutrition secured households. In the USA, for instance, processed FV (frozen, canned or dried) contributed 35 % of dietary fibre, 62 % of vitamin E, 51 % of vitamin C, 40 % of folate and 25 % of vitamin A to diets^(15)^. This affirms that the possibilities of processed FV not only lower post-harvest losses but also contribute to improving the nutritional status of individuals who otherwise would have been malnourished. There have been concerns about processed foods, and indeed some processed foods have been widely documented to have direct linkages with adverse health outcomes such as obesity and other non-communicable diseases, including in sub-Saharan Africa^(14,35–37)^. Therefore, it is vital to make clear distinctions between processed foods such that consumers can make healthy choices. Classification of the healthiness of processed foods by their degree of processing, which is subjective, does not provide a helpful approach for consumers^(13)^. Instead, characterisation of healthiness or otherwise based on their nutrient content combined with some agreed benchmarks for salt, fat, sugar, food additives and calorie quality can be a helpful approach to adopt^(14)^.

Already most consumers perceive the prices of FV as expensive. In the USA, low-income households allocated a larger share of their food budget to purchase fresh FV^(38)^. Yet, the price of canned vegetables was 20 % cheaper than fresh-packaged vegetables, with both having similar nutrient content. The price of canned fruits was competitive to fresh fruits with comparable nutrients in some cases. In addition, both processed FV provided longer shelf life, presenting an excellent avenue for low-income and poor households to afford FV to meet their nutritional requirements year-round^(39)^. However, the prices of the processed FV in this study were high relative to the fresh FV to the extent that their inclusion into diets increased the cost for infants aged 6–11 months. On the other hand, the increase in diet cost for such children group is not surprising because, during such infant stage, careful and appropriate complementary feeding is critical to ensure healthy growth, as nutrient demands are high^(40–42)^. Yet, food choices for these infants are limited in quality and quantity^(42)^ compared to older children and adults. For instance, in Tanzania, current complementary feeding practices are not able to ensure adequate nutrition for infants aged 6–11 months, requiring the need for nutrition interventions to improve nutritional status^(43)^. Interventions such as including nutritious processed FV used in this study into diets could provide adequate nutrition for infants – which, however, would increase diet costs (as evidenced in this study) due to the peculiar nature of infants in ensuring adequate nutrition.

### Shelf life and acceptability of processed fruits and vegetables

The objectives of this study did not include determining the shelf life and acceptability of processed FV; however, it is essential to discuss these themes as they are imperative if processed FV are to be integrated into diets. According to Miller and Knudson^(39)^, in selecting foods, especially by low-income households, their shelf life constitutes one of the determining factors. This is because they can rely on such foods for a more extended period to meet their nutritional needs. Equally, through sensory evaluation, consumers can gauge the acceptability or otherwise of foods^(44)^. A shelf life study performed for CAJ showed 3 months storage capacity with excellent acceptability by consumers^(45)^. Regarding the two fruit nut bars developed within the FruVaSe project, panellists largely accepted both, although the panellists were not the individuals in this study. Shelf life studies were not done on the fruit nut bars; however, as the water content of the final products was 8 %–10 %, perishability processes have been slowed down^(20)^. In a study conducted by Pato Dickson Innocensia^(11)^ in Morogoro, Tanzania, processed cassava leaves were acceptable by consumers compared to fresh cassava leaves. Consumers were also willing to pay for the processed cassava leaves at the prevailing price, indicating that consumers are ready to pay for a premium price on processed vegetables. However, the study was conducted in urban Morogoro, while the present study focused on rural areas. Also, results from sensory studies performed on processed cassava leaves in Rwanda showed overall likeness and acceptability for the product^(46)^. The DCL used in this study had prolonged longevity and were suitable for year-round consumption^(25)^. In addition, in Uganda, similar solar-dried cowpea leaves were acceptable in taste, flavour, and texture and had a stable storage form that could be well marketed for consumption^(44)^. According to Tepe et al.^(47)^, there is a demand and market for processed FV in East Africa. The trend shows that consumers have general acceptability for processed FV. They can provide the longevity needed to help bridge seasonality nutrient gaps, especially for vulnerable groups like children and women.

Although affordability of diets was not calculated in this study, it is assumed that in Morogoro, households may be able to afford the modelled diets but may have to commit a large share of their income as a food budget. However, in Mtwara, the affordability of the diets might be a considerable challenge. With the level of development in Morogoro, infrastructure-wise, and easy accessibility to motorable road networks and urban areas, it could provide households the opportunity of ease of access to diverse and inexpensive but nutritious foods on the market. Additionally, in Morogoro, households can easily engage in off-farm income activities to fetch them extra income to theoretically afford healthy diets. In contrast, in Mtwara – specifically the communities surveyed – road networks are bad with public transport to nearby urban areas scheduled at specific times. This could present a situation where food supply and availability could be limited to only those produced in the communities, consequently limiting food diversity and accessibility to other nutritious foods. Again, off-farm income ventures could be restricted as well, limiting income generation to purchase nutritious foods. On the other hand, improved access to markets – measured by off-farm income and short distance to markets – have been shown to be associated with better household nutrition^(48)^.

### Policy implications and areas for future research

First, investment in science and technology is essential to develop more processing innovations that help process fresh FV and similarly fresh nutritious yet perishable foods such as milk and meat into nutritious products. Also, appropriate channels for transferring these processing innovations to communities where these foods are produced should be developed. This would help farmers/households and small and medium enterprises (SME) process their FV to attract extra income^(14)^. Second, interventions such as subsidies must be provided to SME that engage in FV processing to make processed FV cheaper for the poor^(14)^. Third, nutritious processed FV must be integrated into nutrition guidelines and programmes. For instance, the importance of nutritious processed FV to diets, particularly during off-availability periods of fresh FV, can be included in nutrition education to women and/or caregivers. Equally, processed FV should be introduced into children’s diets, especially in school feeding and other nutrition programmes. They should be educated as well on their importance for healthy development.

Furthermore, similar studies must be performed in different geographical settings to show the contribution of processed FV to households’ diets. Additionally, modelling diets with highly processed FV or other foods in general – as this study was performed with minimally processed FV only – should be done to assess their impact on the rural poor in terms of nutrients and costs. Since the modelled diets are hypothetical, studies on their acceptability should be evaluated to determine whether households will accept diets as modelled or require fine-tuning or reject them^(17)^.

### Study limitations

To the best of our knowledge, the present study is the first to model diets by integrating processed FV to assess their impact in rural communities, where FV production is predominant, especially in Africa. Despite this, the following limitations were identified for this study.

First, there was a high attrition rate for the traders sampled in Morogoro during the second survey. This means data points on availability and prices of some food items might have been missed during the second survey, which could impact the diet cost. Only the foods identified on the market were used for analysis. Foods from households’ production that may be consumed but are not available on the market were not considered and thus led to the neglect of some foods in the models. However, the foods identified from the 24-h recalls but not found on the market were few. Also, the nutrients from all foods used for modelling were based on their raw form; meanwhile, nutrient losses would be expected during food preparation which might further reduce nutrient intake more than reported in the results. The 24-h constraints were based on qualitative recalls instead of quantitative, neglecting the inter-individual portion sizes of food intake. Usage of the qualitative 24-h recall is nonetheless consistent with the FGD, which is also qualitative. Furthermore, the sample sizes adopted for the 24-h constraints were small, especially for the children, which could limit the variability in foods consumed in the study areas to approximate correct dietary habits for modelling. Except for the fruit nut bars, the nutrient contents of the processed FV are based on food composition tables from elsewhere, which could result in under- or overestimation of the potentials of processed FV in local diets. Again, the actual pricing of the processed FV was not determined, but rather prices from similar products were used, which can bias the results of the potentials of processed FV in terms of diet cost reduction. The processed FV were not tested on the specific individuals of the study to understand their acceptability or otherwise for the products. However, as discussed above, there is a general trend for the likeness and acceptability of processed FV. Lastly, this study did not gauge the acceptability and affordability of the modelled diets with processed FV, especially when the models are considered hypothetical. Therefore, this creates a challenge as to whether the consumption of these diets could be actualised for the individuals. Moreover, for households to access such nutritious diets, there should be the financial will. As it stands now, this study is unable to assess in actual terms whether households have the financial will to buy such nutritious diets.

## Conclusion

This present study sought to model standard diets for children and women in rural Tanzania and assess the impact of processed FV to reduce diet costs and ensure nutritional adequacy. The addition of processed FV to diets reduced diet costs except for infants aged 6–11 months, where the cost went up. On nutritional adequacy, the integration of processed FV was able to fill up the micronutrient gaps that persisted in the standard diets for children and women, except in some cases for infants aged 6–11 months, where some of the micronutrient gaps were not fully bridged. Overall, processed FV could provide a feasible avenue to ensure nutritious but cheap diets for households, especially the poor, year-round. Hence, interventions that would spur the processing of FV into nutritious and affordable products should extensively be pursued at international and national levels. Equally, these nutritious processed FV must be integrated into dietary guidelines and nutrition programmes to address the consumption of poor diets. In addition, an unambiguous definition of what ‘nutritious processed foods’ are in general is needed in dietary guidelines to guide consumers to make healthy food choices.

## Supplementary material

For supplementary material accompanying this paper visit https://doi.org/10.1017/S1368980022000982. click here to view supplementary material
