# Supplementary material for: Are processed fruits and vegetables able to reduce diet costs and address micronutrient deficiencies? Evidence from rural Tanzania
Source: Public Health Nutr. 2022 Apr 25;25(9):2637–50. doi: 10.1017/S1368980022000982 (PMC9991728; doi:10.1017/S1368980022000982)
Supplement: Supplementary file 1 [file S1368980022000982sup001.pdf]

Table S1: Weekly minimum and maximum constraints of foods (number of times a food is consumed per week) from food group discussion and 24-hour dietary recall for infants 6-23 months, school-aged children 6-13 years and women15-49years in Mtwara, Tanzania.

Food	FGD						24-hour recall					
	6-23mo.		6-13years		Women		6-23mo.		6-13years		Women	
	Min.	Max.	Min.	Max.	Min.	Max.	Min.	Max.	Min.	Max.	Min.	Max.
Bread	0	14	0	14	0	14	0	0	0	0	0	7
Maize flour	0	14	0	14	0	14	7	14	0	14	7	14
Millet	0	7	0	7	0	7	0	0	0	0	0	0
Rice	0	14	0	14	0	14	0	14	0	14	0	14
Sorghum	0	7	0	7	0	7	0	0	0	0	0	0
Noodles	0	7	0	7	0	7	0	0	0	0	0	0
Wheat flour	0	7	0	7	0	7	0	0	0	0	0	7
Potato	0	7	0	7	0	7	0	0	0	0	0	0
Sweet potato	0	7	0	7	0	7	0	0	0	0	0	7
Beans	0	7	0	7	0	7	0	7	0	0	0	7
Cowpeas	0	0	0	0	0	0	0	0	0	7	0	0
Groundnut	0	0	0	0	0	0	0	0	0	0	0	7
Beef	0	7	0	7	0	7	0	7	0	0	0	7
Offals	0	7	0	7	0	7	0	0	0	0	0	0
Small-dried fish	0	14	0	14	0	14	0	7	0	7	0	14
Dried fish	0	14	0	14	0	14	0	0	0	0	0	14
Fish	0	14	0	14	0	14	0	14	0	14	0	14
Salted fish	0	14	0	14	0	14	0	0	0	0	0	14
Smoked fish	0	14	0	14	0	14	0	0	0	0	0	14
Octopus	0	0	0	0	0	0	0	0	0	0	0	0
Egg	0	7	0	7	0	7	0	0	0	0	0	7
Powdered milk	0	7	0	7	0	7	0	0	0	0	0	0
Milk	0	7	0	7	0	7	0	0	0	0	0	0
Eggplant	0	14	0	14	0	14	0	0	0	0	0	0
Onion	0	14	0	14	0	14	3	14	0	14	7	14
Peas	0	7	0	7	0	7	0	0	0	0	0	0
Banana	0	14	0	14	0	14	0	0	0	0	0	0
Baobab	0	7	0	7	0	7	0	0	0	0	0	7
Coconut	0	7	0	7	0	7	0	0	0	0	0	0
Coconut milk	0	0	0	0	0	0	0	0	0	7	0	0
Lemon	0	7	0	7	0	7	0	0	0	0	0	0
Lemon juice	0	0	0	0	0	0	0	0	0	0	0	0
Mango juice	0	7	0	7	0	7	0	0	0	0	0	0
Mango	0	0	0	0	0	0	0	7	0	7	0	7
Orange juice	0	7	0	7	0	7	0	0	0	0	0	0
Orange	0	7	0	7	0	7	0	0	0	0	0	0
Passion juice	0	7	0	7	0	7	0	0	0	0	0	0
Peach juice	0	0	0	0	0	0	0	0	0	0	0	0
Pineapple juice	0	7	0	7	0	7	0	0	0	0	0	0

Tomato	0	14	0	14	0	14	0	14	0	14	7	14
Coconut oil	0	0	0	0	0	0	0	0	0	0	0	0
Sunflower cooking oil	0	14	0	14	0	14	0	14	0	14	7	14
Biscuit	0	7	0	7	0	7	0	7	0	0	0	7
Chewing gum	0	7	0	7	0	7	0	0	0	0	0	7
Cookies	0	7	0	7	0	7	0	7	0	0	0	7
Mandazi	0	7	0	7	0	7	0	7	0	7	0	7
Cake	0	7	0	7	0	7	0	7	0	0	0	7
Sugar	0	14	0	14	0	14	3	14	0	14	7	14
Sweets	0	7	0	7	0	7	0	7	0	0	0	7
Salt	7	14	7	14	7	14	3	14	3	14	7	14
Baking powder	0	7	0	7	0	7	0	0	0	0	0	7
Chili sauce	0	7	0	7	0	7	0	0	0	0	0	0
Garlic	0	7	0	7	0	7	0	0	0	0	0	0
Ginger	0	14	0	14	0	14	0	0	0	0	0	0
Tomato paste	0	7	0	7	0	7	0	0	0	0	0	0
Cola (Adam)	0	0	0	0	0	0	0	0	0	0	0	0
Ginger drink	0	7	0	7	0	7	0	0	0	0	0	0
Soda	0	7	0	7	0	7	0	0	0	0	0	0
Coffee	0	0	0	0	0	0	0	0	0	0	0	0
Energy drink	0	7	0	7	0	7	0	0	0	0	0	0
Malt	0	0	0	0	0	0	0	0	0	0	0	0
Tea	0	14	0	14	0	14	0	7	0	14	3	14
N			20 women				12		11		109	

Table S2: Weekly minimum and maximum constraints of foods (number of times a food is consumed per week) from food group discussion and 24-hour dietary recall for infants 6-23 months, school-aged children 6-13 years and women 15-49 years in Morogoro, Tanzania.

Food	FGD						24-hour recall					
	6-23mo.		6-13years		Women		6-23mo.		6-13years		Women	
	Min.	Max.	Min.	Max.	Min.	Max.	Min.	Max.	Min.	Max.	Min.	Max.
Bread	0	7	0	7	0	7	0	0	0	0	0	0
Maize flour	7	14	7	14	7	14	7	14	7	14	7	14
Maize	0	14	0	14	0	14	0	0	0	0	0	0
Millet	0	0	0	0	0	0	0	0	0	0	0	0
Rice	0	14	0	14	0	14	0	14	3	14	7	14
Noodles	0	7	0	7	0	7	0	0	0	0	0	0
Wheat flour	0	14	0	14	0	14	0	0	0	0	0	0
Potato	0	7	0	7	0	7	0	0	0	0	0	0
Sweet potato	0	7	0	7	0	7	0	0	0	0	0	0
Bambara groundnut	0	0	0	0	0	0		0	0	0	0	0
Beans	0	14	0	14	0	14	0	7	0	14	0	14
Cowpeas	0	7	0	7	0	7	0	7	0	0	0	7
Groundnut	0	7	0	7	0	7	0	0	0	0	0	0
Peas	0	0	0	0	0	0	0	0	0	0	0	0

Soybean	0	0	0	0	0	0	0	0	0	0	0	0
Liver	0	7	0	7	0	7	0	0	0	0	0	7
Beef	0	14	0	14	0	14	0	7	0	7	0	7
Offals	0	7	0	7	0	7	0	0	0	0	0	7
Small-dried fish	0	14	0	14	0	14	0	7	0	14	0	7
Fish	0	14	0	14	0	14	0	7	0	14	0	14
Salted fish	0	14	0	14	0	14	0	0	0	0	0	14
Shrimp	0	7	0	7	0	7	0	0	0	0	0	0
Egg	0	14	0	14	0	14	0	0	0	0	0	0
Cabbage	0	7	0	7	0	7	0	0	0	0	0	0
Chinese	0	7	0	7	0	7	0	0	0	0	0	0
Carrot	0	14	0	14	0	14	0	0	0	0	0	0
Cucumber	0	14	0	14	0	14	0	0	0	0	0	0
Eggplant	0	14	0	14	0	14	0	0	0	0	0	0
Green pepper	0	14	0	14	0	14	0	0	0	0	0	0
Amaranth	0	14	0	14	0	14	0	0	0	7	0	7
Cassava leaves	0	14	0	14	0	14	0	0	0	7	0	7
Pumpkin leaves	0	14	0	14	0	14	0	7	0	7	0	7
Sweet potato leaves	0	14	0	14	0	14	0	7	0	7	0	7
Okra	0	14	0	14	0	14	0	7	0	0	0	7
Onion	7	14	7	14	7	14	3	14	7	14	7	14
Pumpkin	0	14	0	14	0	14	0	0	0	0	0	0
Radish	0	0	0	0	0	0	0	0	0	0	0	0
Avocado	0	14	0	14	0	14	0	0	0	0	0	0
Banana	0	14	0	14	0	14	0	7	0	7	0	0
Black currant juice	0	7	0	7	0	7	0	0	0	0	0	0
Coconut	0	14	0	14	0	14	0	0	0	7	0	0
Coconut milk	0	0	0	0	0	0	0	7	0	7	0	14
Lemon	0	14	0	14	0	14	0	0	0	7	0	0
Lemon juice	0	0	0	0	0	0	0	0	0	0	0	0
Mango juice	0	7	0	7	0	7	0	0	0	0	0	0
Mango	7	14	7	14	7	14	0	0	0	0	0	0
Orange juice	0	7	0	7	0	7	0	0	0	0	0	0
Orange	0	7	0	7	0	7	0	0	0	7	0	0
Papaya	0	7	0	7	0	7	0	0	0	0	0	0
Passion fruit	0	7	0	7	0	7	0	0	0	0	0	0
Passion juice	0	7	0	7	0	7	0	0	0	0	0	0
Pineapple	0	7	0	7	0	7	0	0	0	0	0	0
Pineapple juice	0	7	0	7	0	7	0	0	0	0	0	0
Tamarind	0	0	0	0	0	0	0	0	0	0	0	0
Tangerine	0	14	0	14	0	14	0	0	0	0	0	0

Tomato	7	14	7	14	7	14	0	14	0	14	3	14
Watermelon	0	7	0	7	0	7	0	0	0	0	0	0
Sunflower cooking oil	0	14	0	14	0	14	3	14	7	14	7	14
Biscuit	0	7	0	7	0	7	0	7	0	7	0	0
Chewing gum	0	0	0	0	0	0	0	0	0	0	0	0
Mandazi	0	7	0	7	0	7	0	7	0	7	0	0
Sugar	0	14	0	14	0	14	7	14	0	14	3	14
Sweets	0	7	0	7	0	7	0	7	0	7	0	0
Baking powder	0	0	0	0	0	0	0	0	0	0	0	0
Chili sauce	0	0	0	0	0	0	0	0	0	0	0	0
Garlic	0	14	0	14	0	14	0	0	0	0	0	0
Ginger	0	14	0	14	0	14	0	0	0	0	0	0
Salt	0	14	0	14	0	14	7	14	7	14	7	14
Pepper	0	7	0	7	0	7	0	0	0	0	0	0
Tomato paste	0	0	0	0	0	0	0	0	0	0	0	0
Cola (Adam)	0	0	0	0	0	0	0	0	0	0	0	0
Strawberry beverage	0	0	0	0	0	0	0	0	0	0	0	0
Energy drink	0	0	0	0	0	0	0	0	0	0	0	0
Malt	0	7	0	7	0	7	0	0	0	0	0	0
Tea	0	7	0	7	0	7	0	0	0	7	3	14
N			20 women				24		41		183	

Table S3: Scoring benchmarks for determining the 24-hour constraints (number of times a food is consumed per week) for infants 6-23 months, school-aged children 6-13 years and women 15-49 years in Mtwara and Morogoro, Tanzania.

Mtwara								
6-23 months			6-13 years			Women		
Score	Minimum	Maximum	Score	Minimum	Maximum	Score	Minimum	Maximum
0.0 – 1.0	0	0	0.0 – 1.0	0	0	0.0 – 22.5	0	0
1.5 – 3.0	0	7	1.5 – 3.0	0	7	23.0 – 54.5	0	7
3.5 – 6.0	0	14	3.5 – 6.0	0	14	55.0 – 109	0	14
6.5 – 8.0	3	14	6.5 – 8.0	3	14	109.5 – 163.5	3	14
8.5 – 12.0	7	14	8.5 – 11.0	7	14	164.0 – 218.0	7	14
N	12		N	11		N	109	
Morogoro								
6-23 months			6-13 years			Women		
Score	Minimum	Maximum	Score	Minimum	Maximum	Score	Minimum	Maximum
0.0 – 2.0	0	0	0.0 – 4.5	0	0	0.0 – 25.0	0	0
2.5 – 8.0	0	7	5.0 – 10.5	0	7	26.0 – 91.5	0	7
8.5 – 12.0	0	14	11.0 – 20.5	0	14	92.0 – 183.0	0	14
12.5 – 18.0	3	14	21.0 – 30.5	3	14	184.0 – 274.5	3	14
18.5 – 24.0	7	14	31.0 – 41.0	7	14	275.0 – 366.0	7	14
N	24		N	41		N	183	

Table S4: Food items of eight food groups identified on the market from 33 traders and their average prices (in TZS) per 100g edible weight across the plenty and lean seasons in Mtwara, Tanzania.

Food	Plenty (TZS)	Lean (TZS)	Yearly (TZS)
**Cereals, roots, and tubers:**			
Bread	40	40	40
Maize flour	161.7	118.4	141.6
Millet	--	350	350
Rice	193.9	200.5	197.4
Sorghum	--	266.7	266.7
Noodles	329.6	322.8	325.6
Wheat flour	147.9	160	152.6
Potato	115.4	133.3	119.9
Sweet potato	56.7	56.7	56.7
Average price	***149.3***	***183.2***	***183.4***
**Pulses and nuts:**			
Beans	217.1	182.8	195.8
Cowpeas	160	--	160
Groundnut	--	200	200
Peas	--	80	80
Average price	***188.6***	***154.3***	***158.9***
**Animal source foods**			
Beef	600	600	600
Offals	600	600	600
Small- dried fish	1037.5	800	956.4
Dried fish	600	500	533.3
Fish	1569.7	840	1377.7
Salted fish	400	333.3	350
Smoked fish	2090.9	2272.2	2157.72
Octopus	1200	--	1200
Egg	1135.6	1521.2	1283.9
Powdered milk	4250	2000	3500
Milk	3125	1816.7	2470.8
Average price	***1509.9***	***1128.3***	***1366.4***
**Vegetables:**			
Eggplant	--	100	100
Onion	301.84	91.6	238.8
Tomato	193.1	430.6	253.7
Tomato paste	158.6	809.5	437.6
Average price	***217.9***	***357.9***	***257.5***
**Fruits:**			
Banana	--	40	40
Baobab	307.8	600	380.9
Coconut	141.1	167.7	159.8
Lemon	--	150.6	150.6
Mango	137.6	--	137.6
Orange	--	48.1	48.1
Average	***195.5***	***201.3***	***152.8***
**Sweets and Beverages:**			
Biscuit	470	505.0	486.4

Chewing gum	1184.9	366.7	892.7
Cookies	818.9	818.9	818.9
Mandazi	371.7	600	463.0
Cake	285.2	285.2	285.2
Sugar	281.8	280.4	281.0
Sweets	1086.4	680	904.2
Lemon juice	133.3	177.8	160
Mango juice	214.3	197.8	209.3
Orange juice	214.9	192.7	208.1
Pineapple juice	183.2	193.3	186.8
Passion juice	164.6	160.6	163.2
Peach juice	175	--	175
Energy drink	194.3	200	195.4
Malt	233.3	150	191.7
Tea	1687.7	1478.3	1595.8
Cola (Adam)	166.7	166.7	166.7
Ginger drink	170.1	170.1	170.1
Soda	183.3	183.3	183.3
Coffee	1300	4400	3160
Average price	***475.9***	***589.8***	***544.8***
**Oils and fats:**			
Sunflower cooking oil	412.8	422.1	417.7
Coconut oil	652.2	652.2	652.17
Coconut milk	3333.3	3333.3	3333.3
Average price	***1466.1***	***1469.2***	***1467.7***
**Spices and condiments:**			
Salt	88	78.3	82.7
Garlic	1539.9	286.0	1181.7
Ginger	471.8	350	410.9
Baking powder	583.3	500	571.4
Chili sauce	179.7	169.2	171.7
Average price	***572.5***	***276.7***	***483.7***

$1 = Tsh 790.5 PPP for private consumption

Table S5: Food items of eight food groups identified on the market from 17 traders and their average prices (in TZS) per 100g edible weight across the plenty and lean seasons in Morogoro (n= traders), Tanzania.

Food	Plenty (TZS)	Lean (TZS)	Yearly (TZS)
**Cereals, roots, and tubers:**			
Bread	329.1	310.6	318.3
Maize flour	120.5	148.6	135.6
Maize	112.9	130.9	127.3
Millet	--	300	300
Rice	149.3	170	161.7
Noodles	294.4	305.2	302.1
Wheat flour	130.6	160	148.2
Potato	118.2	123.4	120.1
Sweet potato	124.5	--	124.5
Average	***172.4***	***206.1***	***193.1***
**Pulses and nuts:**			
Bambara groundnut	--	350	350
Beans	220.3	278.2	255.7

Cowpeas	200.4	236	230.1
Groundnut	276.4	373.3	324.9
Peas	--	303.0	303.0
Soybean	--	200	200
Average	***232.4***	***290.1***	***277.3***
**Animal source foods**			
Liver	803.2	800	801.6
Beef	595.2	600	597.6
Offals	398.4	400	399.2
Small- dried fish	651.7	760.9	749.7
Fish	918.4	918.4	918.4
Salted fish	1431.2	1362.5	1379.7
Shrimp	1020.4	838.6	849.9
Egg	678.9	845.7	804
Average	***812.2***	***815.8***	***812.5***
**Vegetables:**			
Cabbage	151.7	79.4	120.7
Chinese	128.4	--	128.4
Carrot	436.9	349.8	418.2
Cucumber	157.9	134.2	146.1
Eggplant	146.1	119.2	135.8
Green pepper	322.8	306.6	320.2
Amaranth	96.8	60.9	76.6
Cassava leaves	177.2	47.2	121.5
Pumpkin leaves	117.7	75.9	103.8
Sweet potato leaves	119.4	55.3	87.3
Okra	283.2	129.1	217.2
Onion	279.9	380.5	338.75
Pumpkin	--	79.4	79.4
Radish	123.7	--	123.7
Tomato	222.1	268.9	246.9
Tomato paste	--	161.9	161.9
Average	***197.4***	***160.6***	***176.7***
**Fruits:**			
Avocado	207.1	26.7	146.9
Banana	105.5	93.6	100.4
Coconut	135.2	344.5	252.9
Lemon	129.4	205.3	180
Mango	94.5	77.5	91.7
Orange	85.4	57.1	77.3
Papaya	98.5	98.3	98.4
Passion fruit	303.6	201.9	245.5
Pineapple	80.7	--	80.7
Tamarind	--	200	200
Tangerine	173.6	--	173.6
Watermelon	28	--	28
Average	***131.1***	***144.9***	***139.6***
**Sweets and Beverages:**			
Biscuit	524.2	426.3	458.9
Chewing gum	1250	--	1250
Mandazi	264.5	--	264.5
Sugar	248.3	264.5	270.2

Sweets	809.9	937.9	901.4
Black currant juice	138.9	--	138.9
Lemon juice	166.7	--	166.7
Mango juice	195	188.9	190.5
Orange juice	126.3	128.5	126.9
Passion juice	132.5	--	132.5
Pineapple juice	131.3	--	131.3
Cola (Adam)	142.9	--	141.9
Strawberry beverage	141.7	--	141.7
Energy drink	139.9	174.2	165.1
Malt	158.3	175	169.5
Tea	1750	3030.3	2578.4
Average price	***395.0***	***665.7***	***451.78***
**Oils and fats:**			
Sunflower cooking oil	412.6	331.8	362.1
Coconut milk	--	1538.5	1538.5
Average price	***412.6***	***935.2***	***950.3***
**Spices and condiments:**			
Baking powder	--	853.9	853.9
Chili sauce	--	163.4	163.4
Garlic	949.1	--	949.1
Ginger	492.5	400	488.8
Salt	72.7	80	78.2
Pepper	757.7	628.0	683.6
Average price	***568.0***	***425.1***	***536.2***

$1 = Tsh 790.5 PPP for private consumption
